# Optoelectronic Properties of Cold Plasma-Deposited, Oxidized Sn–C Thin Films

**DOI:** 10.3390/ma17020314

**Published:** 2024-01-08

**Authors:** Ewelina Zofia Frątczak, Jacek Balcerzak, Maciej Rogala

**Affiliations:** 1 Department of Molecular Engineering, Faculty of Process and Environmental Engineering, Lodz University of Technology, Wólczańska 213, 93-005 Lodz, Poland; 2 Sub-Department of Physics and Technology of Nanometric Structures, Faculty of Physics and Applied Informatics, University of Lodz, Pomorska 149/153, 90-236 Lodz, Poland

**Keywords:** electronic properties, nanofilaments, cold plasma, conductive atomic force microscopy

## Abstract

We report on investigating the structural and electronic properties of semiconducting and insulating layers produced in a process resembling percolation in a unique cold plasma fabrication method (plasma-enhanced chemical vapor deposition—PECVD). Amorphous carbon–tin films (Sn–C) produced from tetramethyl tin (TMT) with an acoustic-frequency glow discharge in a three-electrode reactor were investigated. The layers, after air exposure, oxidized to SnO_2_/Sn–C. Depending on the coupling capacitance applied to the plasma reactor, the films could be obtained in the form of an amorphous semiconductor or an amorphous insulator. We assume that the semiconductor consists of an internal network of channels auto-organized during deposition. The insulator does not demonstrate any internal structure features. An investigation on conductive filaments creating low-dimensional (LD) nanojunctions in the semiconductor and the location of energetic levels in the insulator was performed. The main parameters of the electronic band structure of the insulating film, such as the transport gap E_G_ (5.2 eV), optical gap E_opt_ (3.1 eV), electron affinity Χ (2.1 eV), and ionization potential J (7.3 eV), were determined. We have demonstrated a simple approach for developing a catalyst candidate consisting of amorphous semiconductor–insulator nanojunctions for (photo)catalytic hydrogen evolution or CO_2_ reduction.

## 1. Introduction

Sunlight’s conversion into renewable energy sources is nowadays one of the hottest topics in nanotechnology. To achieve the maximum efficiency of devices such as solar cells [1] or photoelectrochemical cells [2], fuel batteries [3,4], photovoltaics [5], or catalysts for hydrogen production or CO_2_ reduction [6,7,8], under the influence of solar energy, a photogeneration of electron–hole pairs occurs at semiconductor–metal [9] or semiconductor–insulator [10,11] interfaces. Using LD nano-junctions ensures a sufficiently long transport path for the charge carriers separated on it, which prevents rapid recombination. Due to the appropriate energy band adjustment of the heterojunction, the charge, after passing through the interface, is within the desired energy range. Therefore, the experimental determination of the electronic structure of such junctions is still a challenge from the point of view of basic research and technological solutions. A key role in the development of a new class of semiconductor-based thin films with auto-organized LD heterojunctions toward increased photocatalytic activity is a fast and easy fabrication made possible by the application of cold plasma. The peculiarity of the layers obtained by the application method lies in the possibility of tuning the type of their electrical and optical properties by changing the coupling capacitance during application in cold plasma. This is due to the change in the ions’ energy bombarding the substrate surface. These changes are attributed to the transition between the amorphous semiconductor and amorphous insulator (a-S:a-I) states. The transition can be explained by percolation theory, which assumes that a solid is composed of a set of disordered conductive nodes. With an increasing amount of nodes, the system undergoes the amorphous semiconductor-to-amorphous insulator phase transition, which is explained in [12]. In semiconductors, charge carrier (electron or hole) transport proceeds by extended states or by a hopping mechanism by localized states. In insulators, all states are localized, and the charge carriers can be transported only by hopping. Our knowledge of the electronic structure of these materials and its correlation with the mechanism of electrical conduction is incomplete, and many aspects remain to be clarified in this matter. We attempt to understand the electronic properties to combine them further with the material structure to influence the efficiency of the processes for which the material will be tested. By applying a unique plasma generation method, we have obtained semiconducting layers where a self-assembled internal structure occurs. We have demonstrated an internal structure of layers consisting of semiconducting Sn (oxidized to SnO_2_ by air exposure) channels embedded in an insulating carbon matrix by applying original measurement techniques, such as conductive atomic force microscopy (CAFM) and local conductivity atomic force microscopy (LC-AFM). With a photoinjection technique, we have determined the energy structure of the fabricated layers.

The novelty presented in this work is the creation of self-organizing structures as a consequence of the use of a three-electrode reactor, with the third electrode in a perpendicular arrangement, powered by a plasma generator with a frequency of 40 KHz. At the same time, the combination of unconventional research methods gives results that are interesting in the context of interpretation.

## 2. Materials and Methods

**Figure 1:**
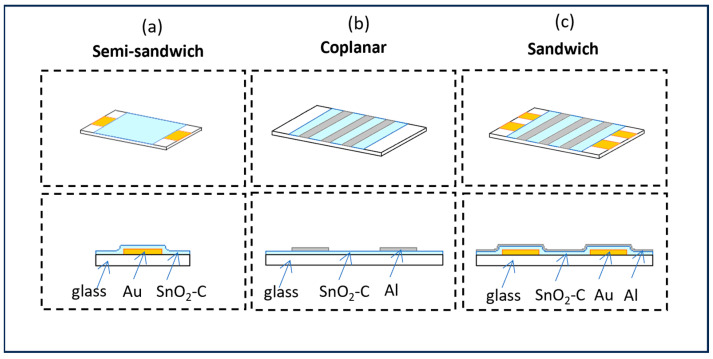
Design of different electrode configurations: (**a**) a semi-sandwich for nanoconductivity measurements, (**b**) coplanar for macroconductivity, (**c**) and sandwich for photoconductivity.

Semiconducting and insulating amorphous SnO_2_–C films were produced from TMT by cold plasma PECVD using an acoustic frequency (af) 40 kHz three-electrode glow discharge reactor. Using a 40 kHz frequency ensures the highest ion density compared with other frequencies, which increases the efficiency and improves the uniformity of the particles. The distance between the main electrodes of the reactor was 2.5 cm, and the third neutral electrode was placed perpendicularly to the main electrodes. The electronic properties of the films strongly depend on the capacity coupling of a neutral electrode on which the films are deposited with a hot electrode sustaining a glow discharge. For a characteristic value of the coupling capacity, specific for given deposition conditions, a minimal variation of the value brings about rapid changes in the DC conductivity of the films [12]. The power supplied to the system was 500 W, and the voltage amplitude values on the neutral electrode were controlled by the coupling capacity between the neutral and hot electrode and amounted to 50 and 1350 V. The amplitude represents the ion impact energy. The flow rate of TMT was 0.3 sccm, and the pressure in the reactor chamber was 4.2 Pa. Semiconducting opaque Sn–C films 120 nm thick and insulating transparent films 130 nm thick were deposited on 0.1 mm thick glass substrates. Three types of electrode geometry (Figure 1) were prepared for conductive atomic force microscopy (CAFM) measurements (Veeco Instruments Nanoscope III with TUNA mode) as well as macroconductivity and photoconductivity measurements (homemade system). The layers were exposed to air, and the following measurement techniques were applied. Measurements of CAFM electrical nanoconductivity were carried out on semiconducting samples prepared on Au electrodes in a semi-sandwich configuration (Figure 1a), macroconductivity was measured in a coplanar geometry (Figure 1b), and photoconductivity measurements were taken on insulating films prepared in sandwich geometry between Au and Al electrodes (Figure 1c). The highly transparent top Al electrodes of known transmittance (typically 10 nm thick) were thermally evaporated perpendicularly to the bottom Au strips forming a sandwich configuration.

The active surface of the electrodes was 4 mm^2^. The photoconductivity measurements were carried out in the 230–600 nm wavelength region. A 150 W xenon lamp and a double prism monochromator were used as the light source. The incident light intensity was measured with a Light Calibrator from The Photon Institute. A quantum efficiency of incident light of 2.2–6.6 × 10^17^ quanta/s*m^2^ illuminated the active surface of the electrodes. Current–voltage dependences were measured in ambient conditions for CAFM (nanoconductivity) and in a vacuum of 0.5 Pa at room temperature for macroconductivity and photoconductivity. Samples were polarized by an external field of 2 × 10^7^ V/m for at least 20 h. The specific conductivity was calculated from ohmic parts of the current–voltage characteristics. For photoconductivity, a step-by-step method was applied [13], and a constant bandwidth of 15 nm was maintained. After fixing a suitable light wavelength, the specimen was illuminated for 20 min. The conductivity of a single filament was measured by a local conductivity mode CAFM (NT-MDT, Moscow, Russia) with an amplifier providing linear characteristics up to 10 nA and logarithmic above this value to represent high dynamics at both low and high currents. Topographic measurement was performed by a contact mode of Atomic Force Microscopy (Veeco AFM, New York, NY, USA). Optical absorption measurements were performed in a wavelength range from 230 to 600 nm (Shimadzu UV-1800 spectrometer, Kyoto, Japan). The XPS analyses were carried out with a Kratos AXIS Ultra spectrometer (Manchester, UK) using monochromatic Al Kα X-rays as an excitation energy source equal to 1486.6 eV. The spectra were collected from several analysis areas of 300 × 700 µm each. The power of the anode was set at 150 W, and the hemispherical electron energy analyzer was operated at a pass energy of 20 eV for all high-resolution elemental spectra. All measurements were performed using a charge neutralizer, and the main carbon peak (C 1s, 285 eV) was used for the final calibration of each spectrum.

## 3. Results

Conductivity and photoconductivity properties were investigated for semiconducting and insulating layers. Both types of active layers were prepared in a cold plasma environment in a three-electrode reactor. The structural and electronic changes indicating an a-S:a-I phase transition were achieved by applying different coupling capacitances between the neutral and active electrodes of the reactor. The insulating layer corresponds to a capacitance of 0 pF, and the semiconducting layer to around 2000 pF.

### 3.1. Nanoconductivity

**Figure 2:**
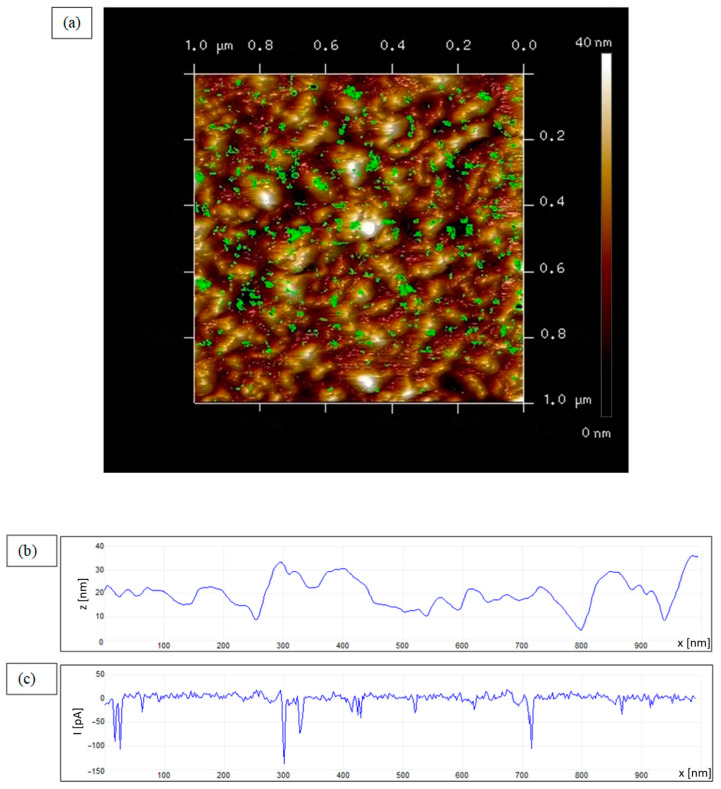
CAFM images of (**a**) 2D distribution of conductive sites in the 3D AFM topographic image indicating the presence of semiconducting nano-filamentary structures in the insulating surroundings; microscope’s magnification 1 × 1 µm. (**b**) Topographical single-line scan and (**c**) corresponding current scan of CAFM.

The existence of semiconducting channels in the layer grown as semiconducting was tested by the conductive mode of AFM (CAFM), while the layer grown as insulating did not show any conductive features. Before performing the conductivity measurements, the layer continuity was checked optically. The layer was continuous and homogeneous. Figure 2a shows a 3D topography image with overlaid conductive structures with arrangements of semiconducting sites on a nonconductive surface 1 × 1 µm. Figure 2b shows a topography scan along one line of 1 μm length. The roughness of the surface is 7.8 nm. The location of the conductive sites is independent of the topographic features of the insulating surface, which is shown in Figure 2c. The calculated area of conductive sites is ~5%.

**Figure 3:**
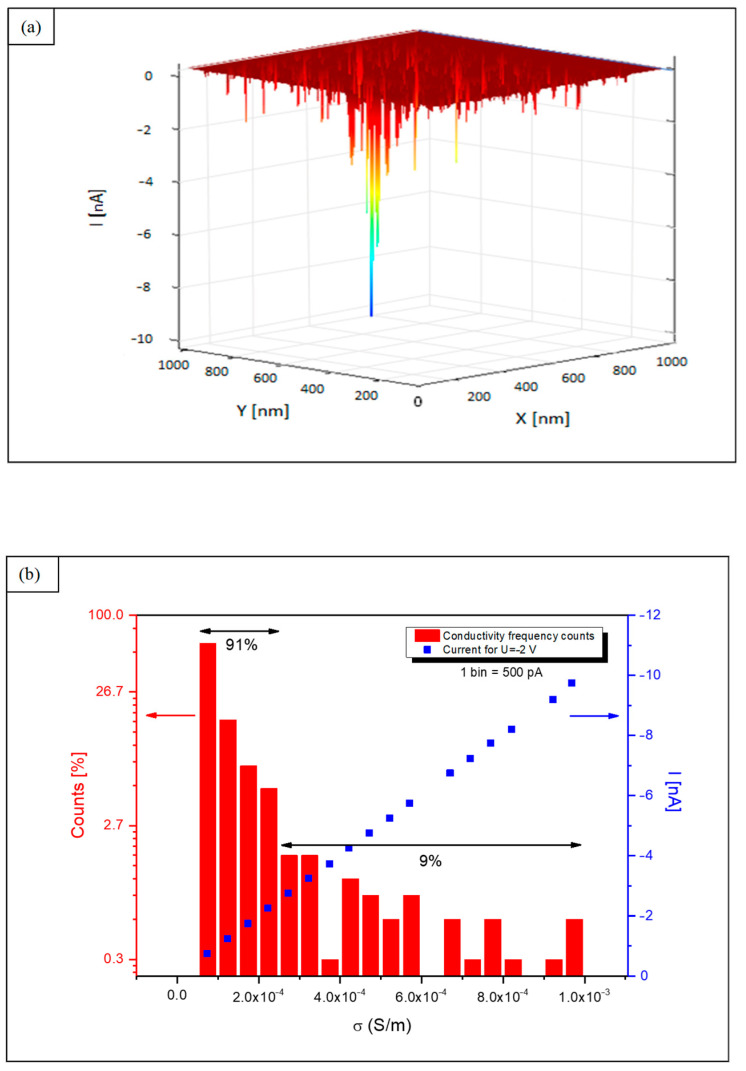
(**a**) A 3D current distribution of conductive sites. (**b**) CAFM nanoconductivity of semiconducting filaments in the semiconducting layer.

The conductive structures were detected in the range of 0.5–2 V of a bias voltage, and the highest currents were recorded at a negative polarization of −2 V at an electric field in the range of 2 × 10^8^ V/m. At higher fields, the currents were not stable. Higher currents were recorded for negative polarization than for positive due to the existence of an asymmetrical junction between electrodes and the active layer (Au electrode—active layer—CAFM Pt/Ir tip). The 3D distribution of currents measured at conductive sites is shown in Figure 3a. In Figure 3b, the conductivity values (red bars) are shown in the histogram, where the most frequent counts (91%) indicate average conductivity, and 9% of counts are attributed to high conductivity from 2.4 × 10^−4^ S/m up to 9.9 × 10^−4^ S/m, respectively. The nanoconductivity values testify to the existence of the semiconducting nature of the channels self-created in insulating surroundings during plasma deposition.

### 3.2. Local Conductivity

**Figure 4:**
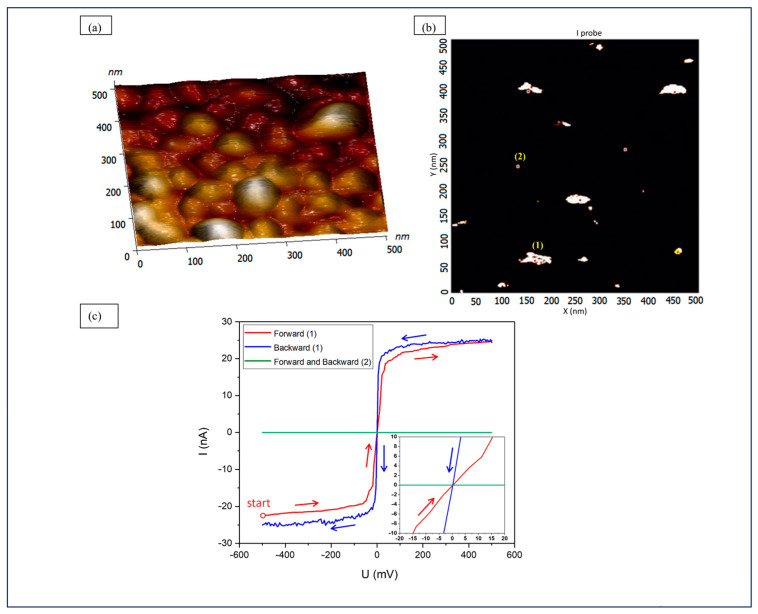
(**a**) Topography and (**b**) conductivity map with I probe scans of the single conductive channel (1) and insulating surroundings (2). (**c**) Forward (from negative to positive voltages) and backward (from positive to negative) scans of the conductive channel (point 1) and isolating surroundings (point 2). Please note that the current converter used for the measurements is logarithmic above 10 nA, which determines the shape of the curve in 10–25 nA range [16].

The local conductivity of one channel was measured by the I probe of LC-CAFM, and its value was 241 S/m, so the roughly calculated conductivity of one channel from the 1 × 1 µm CAFM measurement is very close to this value. Kim et al. reported the conductivity of the SnO_2_ nanowire to be similar and amount to 276 S/m [14], and Kiruthiga gave a conductivity value of 260 S/m for a pure SnO_2_ thin film [15]. The high similarity of local conductivity values for conductive channels in our layers and nanowires from the literature indicates that we can expect SnO_2_ channels in our samples. For comparison, the conductivity of pure tin is 9.2 × 10^6^ S/m. Figure 4 shows the topography (Figure 4a) and the conductivity map of the I probe scan (Figure 4b) with a selected single conductive channel (1) and insulating surroundings (2). Forward (from negative to positive voltages) and backward (from positive to negative) scans of the conductive channel (point 1) and isolating surroundings (point 2) are shown in Figure 4c. The channel exhibits ohmic conductivity (red and blue), and the surroundings show no conductivity at all (green). The discrepancy between the forward (red) and backward (blue) curves at point (1), visible as hysteresis, can result from the higher current flow on the positive voltage side. In consequence, the tip–channel contact improves because contamination on the tip is reduced. Then, the characteristics are reliable and reflect the channel conductivity. Probably due to carbon mass transfer by the CAFM tip at the scanned surface, it was not possible to measure the conductivity of the single smaller channel. The sizes of individual channels were calculated based on CAFM maps of conductive site diameters and range from 20 to 500 nm^2^. It should be noted that the shape of the channels and their surface area may be disturbed by convolution with the shape of the AFM tip; however, AFM well reflects the order of magnitude of the sizes and their large distribution.

### 3.3. Macroconductivity

The macroconductivity of the semiconducting SnO_2_–C composite layer measured in coplanar geometry (Figure 1b) is 3.97 × 10^−3^ S/m, and the nanoconductivity value ranges from 2.4 × 10^−4^ S/m for average currents to 9.9 × 10^−4^ S/m for high currents as described above. Nanoconductivity is approximately one order of magnitude lower than macroconductivity because, for thin-film systems, the conductivity is one order lower than for volumetric systems. We assume that SnO_2_ filaments of the layer are responsible for the macroconductivity properties. Macroconductivity for the insulating film is 3.4 × 10^−12^ S/m, which gives an eight-order difference compared to the semiconductive layer. Such a significant change in conductivity for the two extreme capacity parameters indicates the occurrence of the percolation phenomenon, which was described for Sn–C layers in [12]. However, other phenomena resulting from changes in the chemistry of the plasma phase (i.e., species concentration, ion bombardment, electron temperature) can also influence the difference in conductivity. For both macroconductivity and photoconductivity measurements, the samples were kept in a 0.5 Pa vacuum after air exposure during transfer from the reactor to the measuring chamber.

### 3.4. Atomic Concentration

The sample surface was analyzed by XPS spectroscopy. Wide scans revealed that both insulating and semiconducting deposits are composed of oxygen, carbon, and tin. The atomic concentration of those elements is summarized in Table 1.

**Figure 5:**
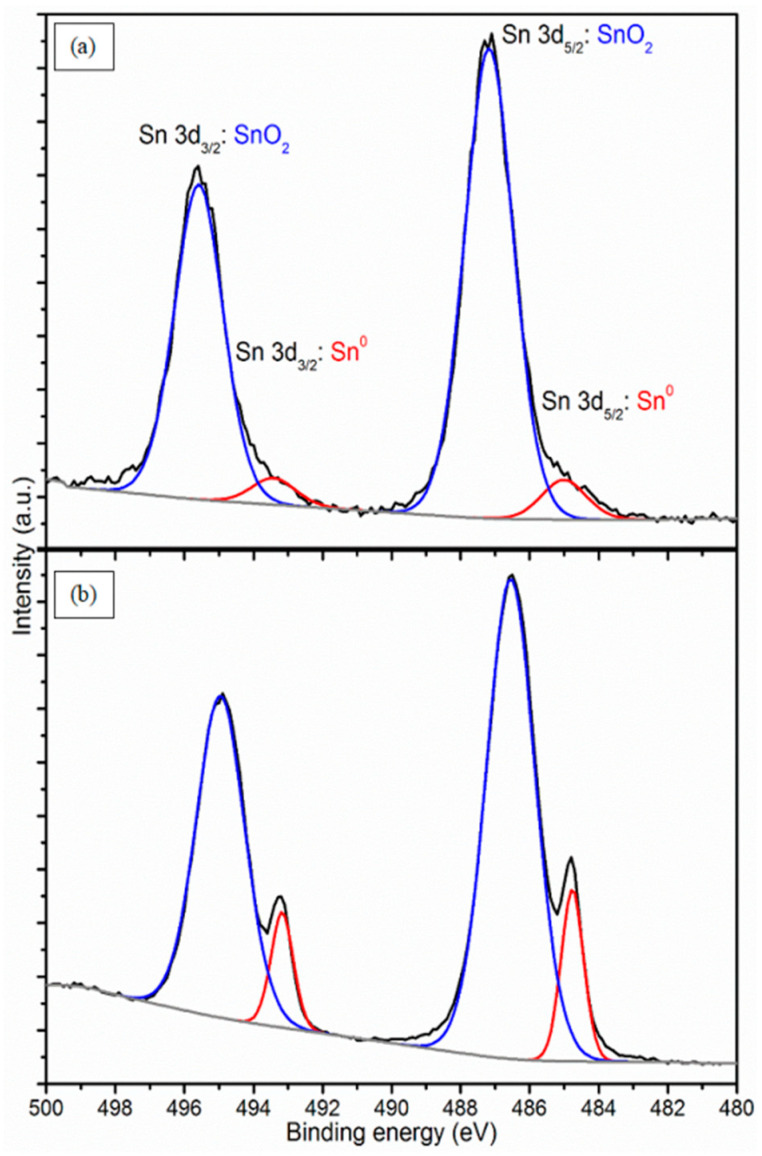
XPS spectrum of Sn 3d orbit-spin doublet: (**a**) insulating SnO_2_–C; (**b**) semiconducting SnO_2_–C. Black line—experimental curve, blue line—fitting of SnO_2_ fraction, red line—fitting of Sn^0^, grey line—background.

It can be noted that the tin contribution in the semiconducting SnO_2_–C sample is 2.5 times greater than for insulating SnO_2_–C. Moreover, the ratio of tin to oxygen for a semiconducting deposit is twice as high as for an insulating one, which in turn is close to the theoretical value of 0.5 for SnO_2_. In Figure 5, 3d XPS high-resolution spectra of Sn are presented. The theoretical ratio of the area of the orbit-spin doublet is equal to d_5/2_:d_3/2_ = 3:2 and, as can be seen in Figure 5, 3d Sn peaks are well fitted with two doubled symmetrical components reflecting tin species. Metallic tin (Sn^0^) is assigned by 3d_5/2_ contributions to 485.0 eV for the insulating sample and 484.8 eV for the semiconducting one. The second, dominant component at 487.2 eV and 486.6 eV can be assigned to Sn^4+^, i.e., SnO_2_, at the surface of the insulating and semiconducting layer, respectively [17]. The main difference between both deposits is the chemical state of tin. The semiconducting sample contains 14.2% metallic Sn, whereas the insulating sample only 7.2%. This can definitely affect the electronic properties of deposited films. The higher Sn atom concentration is responsible for semiconductive properties. The higher carbon concentration in the insulating sample confirms the insulating properties of the carbon matrix. The coefficient expressing the ratio of the tin atomic concentration in the semiconducting sample to the tin atomic concentration in the insulating sample is the same as the coefficient expressing the Sn:O ratio in both samples and equal to 2.4. The oxide formation is proportional to the Sn concentration.

By comparing XPS analysis results with nanoconductivity and local conductivity, we can maintain the assumption that the semiconductive filaments are formed in a self-assembly process from Sn atoms partly oxidized to SnO_2_. The formation of semiconducting, mainly SnO_2_ chains in the insulating C matrix is attributed to the percolation dependent on the coupling capacity applied in the deposition process.

### 3.5. Internal Photoemission (Photoinjection)

Internal photoemission was measured to estimate the electronic structure of the insulating layer, assuming Schottky conduction is limited by contact barriers with electrodes [18]. Photoconductivity measurements were taken in sandwich geometry, where the active layer is placed between Au and Al electrodes (Figure 1c). Determination of the barrier height formed at the metal–layer contact makes it possible to distinguish between bulk and electrode processes. A simple model of photoinjection of electrons and holes from metal to insulator expresses that the sum of the heights of contact barriers for electrons Φ_0e_ and holes Φ_0h_ gives the value of the transport gap E_G_ for the investigated insulator (Table 2).

Φ_0e_ + Φ_0h_ = E_G_ (1)

**Figure 6:**
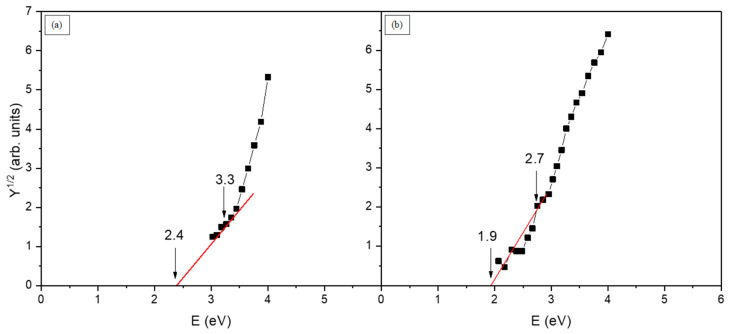
Fowler’s diagram electrode system (Al–film–Au); (**a**) negatively polarized Au, (**b**) positively polarized Au. Applied field was in the range of 10^7^ V/m. The threshold values are given in eV. Black squares represent measurement values.

The photoconductivity expressed as a dependence of the photocurrent quantum yield in the range of photon energy was analyzed based on the Fowler relation:

Y^1/2^ ~ hν − Φ, (2)

where Y is the photocurrent quantum yield, hν is the exciting photon energy, and Φ is the contact barrier height for electrons or holes. Figure 6 shows the photoconductivity of the insulating film in sandwich geometry with asymmetrical electrodes Al–film–Au, presented in Fowler’s coordinate system. The measurements were performed at two opposite polarizations to confirm the existence of the thresholds. Two photoinjection thresholds are distinguished in the plot: the lower threshold corresponding to the energy where the approximated straight line crosses the abscissa, and the upper one—the point from which nonlinearity of the measured curve can no longer be approximated by a straight line assuming the additivity of photoinjection currents. The threshold values depend on the electrode material and are related to the contact barrier heights [13,19]. For a given polarization, one threshold is connected with Au and the other one with Al. Since the work function for Au is higher than for Al, according to the procedure described by J. Tyczkowski and M. Kryszewski [13,20], it may be concluded that the lower threshold is connected with electron photoinjection, whereas the upper one refers to hole photoinjection. Taking into account the fact that the work function for Au is higher than that for Al, in our case, they amount to about 4.6 and 3.8 eV, respectively; the difference in threshold heights for electrons as well as for holes measured for the Au and Al electrodes is about 0.6 eV.

The difference can be explained by the surface oxidation of the Sn component in the active layer and the reduction in the density of initial surface states at the contacts, confirmed by the XPS measurements. A similar effect was also observed for plasma-deposited Ge–C films and organosilicon films, for which air exposure affected the decrease in surface states in comparison to fresh films, and as a result, a difference in threshold heights for electrons and holes appeared [19]. Generally, very small differences in threshold heights for both electrons and holes between the Au and Al electrodes indicate a high density of surface states at the metal–active layer contact [21], as explained in [19]. The value of the barrier height is also related to such parameters as ionization potential or electron affinity (Table 2). Electron affinity Χ and the ionization potential J were calculated according to relations [20]:

Χ = W_M_ − Φ_0e_, (3)

where W_M_*,* is the work function of the metal, and Φ_0e_ is the barrier height for an electron.

J = E_G_ + Χ, (4)

where E_G_ is the transport gap. The calculations for the contact between the metal electrode and insulating SnO_2_–C are consistent with a simple photoinjection model for a metal–insulator contact [22]. The calculation method for electron affinity and ionization potential is presented in Supplementary Material S1.

**Figure 7:**
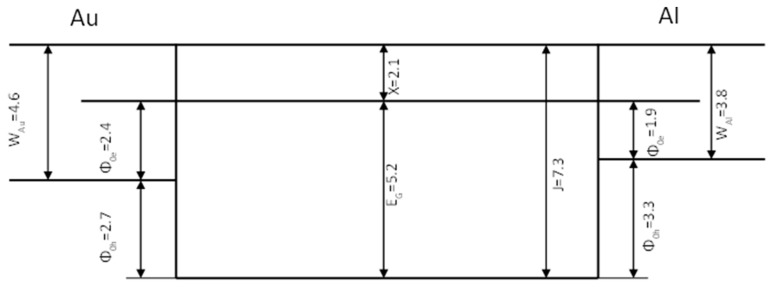
Diagram presenting band structure of the insulating SnO_2_–C film and its contacts with Au and Al. All values are presented in eV.

A model of the electronic band structure for insulating Sn–C films is proposed. In Figure 7, such a model is sketched for oxidized films. Based on the slight conductivity difference for the two directions of the applied field, we can assume that we are dealing with bulk-limited (i.e., Poole–Frenkel) conduction. For contact-limited (Schottky) conduction, the difference should result in about eight orders of magnitude difference in the conductivity measured for two directions of the applied field [18].

### 3.6. Optical Absorption

**Figure 8:**
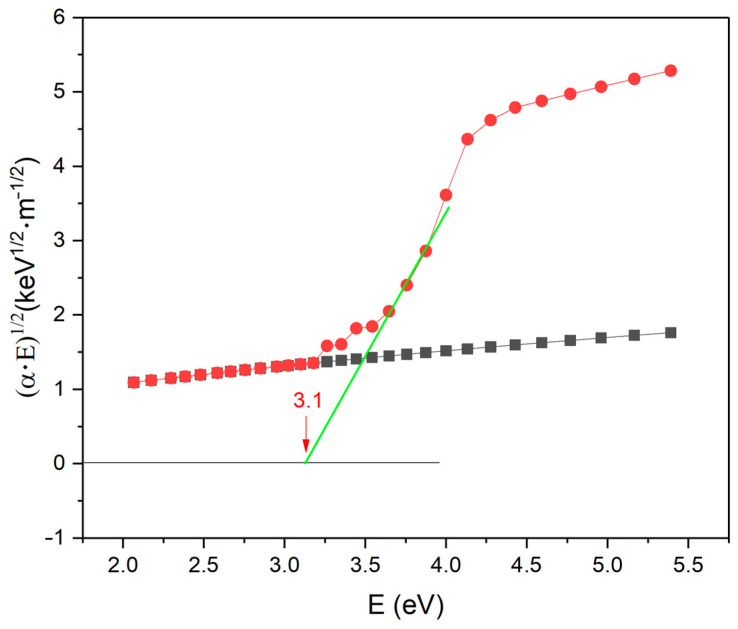
Optical absorption of the films; red points—insulated layer, black points—semiconducting layer. Optical gap marked in coordinates of absorption coefficient vs. photon energy hν according to the Tauc relation (Equation (5) with n = 2). The coefficient B is given in 107 eV^−1^ m^−1^.

Investigation of optical absorption permits the determination of the optical gap and makes it possible to construct a general model of energy levels for insulating SnO_2_–C films, which are transparent. The optical gap E_opt_ has been estimated from UV–Vis absorption spectra by applying the Tauc equation:

α(hν) = B(hν − E_opt_)^n^, (5)

where α is the absorption coefficient, hν the energy of absorbed light, n is a parameter connected with the density-of-states (DOS) distribution, and B is a proportionality factor. This equation, limited to the value n = 2, was originally proposed by Tauc et al. [19,23]. The value of the optical gap is interpreted as a difference between the energy levels of the localized states in the valence and conduction bands, where the electronic transport proceeds by hopping and is lower than the transport gap. Figure 8 depicts the absorption edge for the insulating SnO_2_–C film plotted according to the Tauc relation in Equation (5) with E_opt_ = 3.1 eV, also included in Table 2. The semiconducting film did not show any optical activity because of its opacity.

## 4. Conclusions

In the deposition process, depending on the coupling capacity in the reactor, we obtain semiconductive or insulating layers. Both layers are deposited from TMT and contain metallic Sn. Insulating layers do not demonstrate any internal structure features (CAFM). In both samples, when exposed to air, most of the Sn is oxidized to SnO_2_, as indicated by local conductivity and atomic concentration tests (XPS). For the transparent insulating sample, internal photoemission measurements were used to estimate its electronic structure. The width of the energy gap of 5.2 eV (Table 2) suggests the insulating nature of the layer but due to the content of SnO_2_/Sn (n-type) and the existence of a rather wide optical gap of 3.1 eV, it can be also considered an n-type wide-gap semiconductor.

We have shown that for the two extreme capacitances in the fabrication process, there is an abrupt transition of conductivity associated with an increase in the content of the semiconducting phase in the layer. We attribute this to the observations of the appearance of semiconductive self-assembled filaments in an insulating carbon matrix for semiconductive samples. We explain it with the occurrence of the percolation phenomenon, which has already been described for tin–carbon layers.

The semiconductor–insulator nanojunctions are highly promising structures for application as catalysts for converting sunlight into hydrogen extraction or CO_2_ reduction. Detailed structural and electrical studies of the semiconductor channels and the heterojunctions formed by them with the insulator matrix will be performed as the next step of the research on these materials.

## Figures and Tables

**Table 1:** XPS sample elemental composition.

Sample	O [%at]	C [%at]	Sn [%at]	Sn:O
Semiconducting SnO_2_–C	31.0 ± 0.2	43.1 ± 0.6	25.9 ± 0.8	0.84 ± 0.03
Insulating SnO_2_–C	26.6 ± 0.5	63.3 ± 0.3	10.1 ± 0.3	0.38 ± 0.02

**Table 2:** Main parameters of the electronic band structure of insulating SnO_2_–C film. The differences in values of the thresholds are within the limits of the accuracy of a measurement.

Parameters of Insulating Films	Energy (eV)
Au contact barrier for electrons Φ_0e_	1.9
Au contact barrier for holes Φ_0h_	2.7
Al contact barrier for electrons Φ_0e_	2.4
Al contact barrier for holes Φ_0h_	3.3
Transport gap (Φ_0e_ + Φ_0h_)	5.2
Optical gap	3.1
Electron affinity Χ	2.1
Ionization potential J	7.3

## Data Availability

Data are contained within the article and supplementary materials.

## Supplementary Materials

The following supporting information can be downloaded at: https://www.mdpi.com/article/10.3390/ma17020314/s1, Supplementary Materials S1: Electron affinity and ionization potential calculations. Ref. [22] is cited in the supplementary materials.
