# Supplementary material for: Optoelectronic Properties of Cold Plasma-Deposited, Oxidized Sn–C Thin Films
Source: Materials (Basel). 2024 Jan 8;17(2):314. doi: 10.3390/ma17020314 (PMC10817613; doi:10.3390/ma17020314)
Supplement: Supplementary file 1 [file materials-17-00314-s001.zip › materials-2744763-supplementary.pdf]

Electron affinity and ionization potential calculations

Studies of the barrier heights at a metal-dielectric contact make it possible to calculate the electron affinity:

$$X = W_M - \Phi_{0e},$$

where W_M is the work function of the metal, and Φ_{0e} is the barrier height for an electron. When the energy gap E_G is known, the value of X, in turn, enables estimating other parameters characterizing the energy structure as the ionization potential:

$$J = E_G + X.$$

The calculations for the contact between the metal electrode and insulating SnO_2-C are consistent with a simple photoinjection model for a metal-insulator contact (Caywood 1970). On the base of this model, the barrier to hole injection from the metal into the insulator Φ_{0h} is the ionization energy J minus the metal work function W_M:

$$\Phi_{0h} = J - W_M;$$

the barrier to electron injection Φ_{0e} is W_M minus the electron affinity X:

$$\Phi_{0e} = W_M - X,$$

and the sum of these two barriers, Φ_{0e}, and Φ_{0h}, constitutes the energy gap E_G:

$$\Phi_{0e} + \Phi_{0h} = E_G.$$

Using the determined contact barrier heights for electrons and holes (Table 2) and also the values of the work functions of Au and Al, it was estimated that J = 7.3 eV and X = 2.1 eV. Considering the barrier heights for holes and electrons as well as the values of the electron affinity and ionization potential, an energy levels scheme for SnO_2-C for insulating films can constructed.

Caywood, J.M. Photoemisssion from Metal Contacts into Anthracene Crystals: A Critical Review. *Mol. Cryst. Liquid. Cryst.* **1970**, 12, 1-16, doi: 10.1080/15421407008082756.
